# Effect of timely diagnosis and treatment on growth and body proportionality of children with congenital hypothyroidism

**DOI:** 10.3389/fendo.2025.1713739

**Published:** 2026-01-28

**Authors:** Aditya Kochar, Harvinder Kaur, Devi Dayal

**Affiliations:** 1 Child Growth & Anthropology Unit, Department of Pediatrics, Postgraduate Institute of Medical Education & Research (PGIMER), Chandigarh, India; 2 Pediatric Endocrinology Unit, Department of Pediatrics, Postgraduate Institute of Medical Education & Research (PGIMER), Chandigarh, India

**Keywords:** body disproportion, growth impairment, levothyroxine therapy, Manouvrier’s index, newborn screen (NBS), stunting

## Abstract

**Background:**

Congenital hypothyroidism (CH) is a preventable cause of growth and neurodevelopmental deficits. In India, lack of universal newborn screening (NBS) delays diagnosis. This study examines how treatment timing influences growth, nutrition, and body proportionality in children with CH.

**Methods:**

This cross-sectional study included 66 children with CH, aged under 5 years, receiving Levothyroxine(LT4) therapy for one year. Age and sex-matched 64 healthy children were also enrolled. Children with CH were stratified into early-treated (<3months) and late-treated (>3months) groups. Anthropometric measurements were converted to Z-scores using WHO standards. Body proportionality was assessed using: height–arm span difference, upper segment-to-lower segment (US: LS) ratio, Manouvrier’s Indice Skélique (MIS). MIS classifies skeletal proportions as brachyskelia (short legs, long trunk; score ≤84.9), mesatyskelia (intermediate; 85.0–89.9), and macroskelia (long legs, short trunk; ≥90).

**Results:**

Children with CH had significantly lower height-for-age (HAZ −2.31 ± 1.32 vs. −0.41 ± 0.96), weight-for-age (WAZ −1.98 ± 1.41 vs. −0.36 ± 1.02), and head circumference-for-age (HCZ −1.52 ± 1.28 vs. −0.21 ± 0.89) Z-scores compared with healthy controls (all p < 0.001). Stunting (HAZ < −2 SD) was present in 53% of children, while underweight (WAZ < −2 SD) was observed in 36.4%. Late-treated children had a higher prevalence of severe stunting (67.7% vs. 11.4%), severe underweight (29.0% vs. 2.9%), and microcephaly (35.5% vs. 8.6%) compared with early-treated children (p < 0.05). Disproportionate stature was observed in 71.0% of late-treated children compared with 42.9% of early-treated children (p < 0.05). US: LS ratio and Manouvrier’s Index further confirmed greater skeletal disproportionality in late-treated children compared with early-treated children (p < 0.05).

**Conclusions:**

Delayed treatment in CH significantly impairs growth, increases skeletal disproportionality, and adversely affects neurocranial development. Early initiation of therapy—ideally within the first 3 months—is essential. These findings highlight the need for newborn screening in India.

## Introduction

Thyroid hormones play an essential role in growth, neurodevelopment, and protein metabolism (1). Congenital hypothyroidism (CH), which accounts for a large percentage of cases causing growth and developmental delay, is further subdivided into permanent or transient conditions based on the treatment required. Congenital hypothyroidism most commonly results from thyroid dysgenesis or defects in thyroid hormone synthesis (dyshormonogenesis). Thyroid dysgenesis, including ectopy, athyreosis, and hypoplasia, accounts for approximately 85% of cases and is usually sporadic, whereas central hypothyroidism due to congenital TSH deficiency is rare and often associated with hypopituitarism (2, 3). Left untreated, severe short stature and neurocognitive deficits will ensue. Growth impairment, prolonged jaundice, and vague complaints such as tiredness, cold intolerance, and constipation are among the most common presentations (4). Long-term hypothyroidism, as a result of untreated CH can result in impaired linear growth, translating to an irreversible loss in final adult stature (5). Thyroid hormone deficiency during early life disrupts growth plate function by impairing chondrocyte proliferation, epiphyseal maturation, and endochondral ossification, and delayed initiation of therapy may result in incomplete catch-up growth (6, 7). The prevalence of CH varies worldwide, and Asians have the highest rates. In India, the prevalence is 1 in 722 live births, but the prevalence is higher in high-risk neonates (8–10). Despite its widespread systemic impact, CH is frequently diagnosed late due to the absence of a national neonatal screening program (NBS).

Treatment for CH includes early levothyroxine (LT4) therapy to normalize hormone levels and achieve a euthyroid state, which is important for normal growth. Studies conducted around the world, have shown that starting treatment within the first few weeks of life significantly improves growth trajectories (3, 11–21). LT4 therapy has been shown to improve linear growth, weight, head circumference, and bone maturation in children with CH; however, reported growth outcomes vary across studies, particularly in relation to disease severity and the timing of diagnosis and treatment, suggesting that delayed initiation of therapy may be associated with less optimal growth outcomes (22). Furthermore, children with congenital hypothyroidism missed by newborn screening present later with growth impairment and may demonstrate incomplete catch-up growth despite levothyroxine therapy, with those commencing treatment beyond 30 days of age exhibiting significantly lower weight and height indices, underscoring the lasting consequences of delayed diagnosis (2, 23). However, a few researchers found that earlier diagnosis and LT4 therapy have no significant impact on the final height of patients with CH (24, 25). Several variables, including the first dose of LT4, the age at which TSH normalizes, the timing of screening for CH, and the severity of the disease, may affect the physical growth of children with CH (15, 26). Recent data from a multicenter trial indicate that growth outcomes are comparable across different initial LT4 dosing regimens, provided dosing remains within the recommended range (27).

Although the adverse effects of delayed diagnosis and treatment of congenital hypothyroidism on linear growth are well recognized, most existing studies—largely from newborn screening–detected cohorts—have focused primarily on height outcomes or neurodevelopmental. Data on body proportionality and segmental growth patterns, which may better reflect long-term skeletal disturbances, remain limited, particularly from Indian populations where delayed diagnosis is still common. To address these gaps, the present study was designed to systematically evaluate growth, nutritional status, and body proportionality in children with congenital hypothyroidism, with specific emphasis on the timing of levothyroxine initiation. This approach enables comprehensive assessment of both overall growth impairment and skeletal disproportionality associated with delayed treatment in children with congenital hypothyroidism.

## Methods

This was a hospital-based, cross-sectional observational study conducted at a tertiary care center in North India between July 2023 and December 2024. The study enrolled children diagnosed with CH as per the Indian Society for Pediatric and Adolescent Endocrinology (ISPAE) 2018 guidelines (28, 29), aged less than 5 years, in whom diagnosis and LT4 therapy were initiated before 1 year of age, and who were maintained on adequate treatment (TSH < 5 µU/ml) for at least one year. Children with physical disabilities, dysmorphic features in whom accurate anthropometric measurements were not feasible were excluded. *A priori* sample size calculation was not performed, and all eligible children meeting the inclusion criteria during the study period were included, consecutively. A total of 66 children with CH (male: 43, female: 23) who complied with our inclusion criteria were enrolled from the Pediatric Endocrinology Clinic of the Department. Similarly, 64 age and sex matched controls (male: 38, female: 26) were enrolled from the Growth Clinic of the Department.

Participants were categorized into three groups:

- Group I: Children with CH in whom treatment was started before 3 months of age
- Group II: Children with CH in whom treatment was started between 3 months and 1 year of age
- Group III: Age- and sex-matched healthy controls with no known endocrine or chronic illnesses

The study protocol was approved by the Institutional Ethics Committee (INT/IEC/2023/SPL-860, dated: 18.08.23). Written informed consent was obtained from parents or legal guardians following detailed explanation of study procedures and the voluntary nature of participation. Demographic details, clinical history, and socio-economic status were documented.

Anthropometric evaluations comprised weight, supine length/standing height, BMI, head circumference, MUAC, arm span, crown-rump length, upper segment:lower segment ratio, skinfold thickness, and Manouvrier’s Indice Skelique. All these measurements were conducted using standardized techniques and calibrated instruments in the Growth Laboratory.

Body weight was measured using an electronic weighing scale with a platform (Make: Avery, Capacity: 150 kg, least count: 50 grams). Supine length and Crown-Rump Length (CRL) were measured using an Infantometer (Make: Holtain Limited, UK, least count: 1 mm) for children up to 2 years of age. A Stadiometer (Make: Holtain Limited, Crymych, Dyfed, UK, least count: 1 mm) was used to measure the height in children older than 2 years. Sitting height was measured using a Sitting Height Table (Make: Holtain Limited, UK, least count: 1 mm) to determine the upper segment length in children older than 2 years. Body Mass Index was calculated by dividing weight in kilograms by the square of height in meters. Head circumference and MUAC were measured using a fiberglass tape (least count: 1 mm). Arm span was measured using an arm span measuring device (least count: 1 mm). SFT was measured using a Harpenden Skinfold Caliper (Make: Holtain Limited, UK, least count: 0.2 mm) at two sites: triceps and sub-scapular.

Upper Segment (US) was measured as CRL in children under 2 years and as sitting height in those over 2 years. Lower Segment (LS) was calculated by subtracting CRL from supine length in children under 2 years, and by subtracting sitting height from standing height in older children.

The US: LS ratio was calculated by dividing the upper segment by the lower segment. The normal ratio changes with age—typically 1.7:1 at birth, ~1.3:1 at 3 years, 1.2:1 at 5 years, 1:1 by 9 years, and stabilizing around 0.9:1 in adulthood (30).

Manouvrier’s Indice Skelique (31) was calculated as follows:

- For age <2 years: ((Length − CRL)/CRL) × 100
- For age >2 years: ((Standing height − Sitting height)/Sitting height) × 100

Based on the calculated values, the children were categorized as:

- Brachyskelia (short legs and a long trunk**):** Up to 84.9
- Mesatyskelia (intermediate state**):** 85.0–89.9
- Macroskelia (long legs and a short trunk**):** ≥90

Height and arm span difference was also assessed among children with CH and controls. A difference of > or < 3.5 cm between height and arm span was considered significant disproportionality.

Parents’ height (mother’s and father’s) was recorded once during the study. Mid-parental height (MPH) was calculated using the formula:

(Father’s height + Mother’s height) ± 13/2.

Target height range was defined as MPH ± 6.0 cm.

### Statistical analysis

Categorical variables were presented as counts and percentages. The assessment of data normality was performed using the Kolmogorov–Smirnov test. The choice of statistical tests (parametric or non-parametric) was based on the results of this normality testing. For normally distributed data, the means ± standard deviations were reported; for skewed data, medians and interquartile ranges were reported. The proportionality index (Manouvrier’s Indice Skelique) was computed for body segments. For the comparison, t-tests were used in case of normally distributed data, and the Mann-Whitney U-tests were used in case of skewed data. The paired t-tests as well as Wilcoxon Signed Rank tests applied for time-related variables. Categorical data were summarized by Pearson Chi-square or Fisher’s exact test, where applicable. A p-value < 0.05 was considered as statistically significant; all analyses were conducted using the IBM SPSS Statistics, version 22.0.

Weight for age Z, Height for Age Z score, Head circumference Z score, BMI for age Z-score and MUAC Z-scores, SFT for age Z score, were calculated using WHO MGRS Growth Standards (32, 33).

## Results

The mean age at diagnosis among children with CH was 3.42 months (range: day 3 of life to 11 months). The mean initial LT4 dose was 12.03 ± 6.16 µg/kg/day, and was subsequently titrated to maintain serum TSH within age-appropriate reference ranges; LT4 was administered in tablet form, crushed and dissolved prior to oral administration. The mean age at LT4 treatment initiation was 1.07 ± 0.72 months in the early-treatment group (<3 months) and 6.07 ± 3.36 months in the late-treatment group (>3 months), indicating a statistically significant delay. A parental history of hypothyroidism was documented in three children (4.5%), involving one father and two mothers, none of whom had a history of congenital hypothyroidism. The paternal case and one maternal case were due to autoimmune thyroid disease, while the other maternal case was attributed to iodine deficiency.

### Growth patterns

Children with CH consistently demonstrated lower growth outcomes in both height and weight compared to healthy peers. Among boys, mean height-for-age Z-scores (HAZ) improved from –2.77 at age 1 to –1.91 at age 5, while weight-for-age Z-scores (WAZ) increased from –1.90 to –1.27. Girls showed more pronounced catch-up growth, with HAZ improving from –2.94 to –0.96 and WAZ from –2.22 to –0.89 over the same period (**Table 1**). Despite these gains, growth faltering remained evident. Over half of the cohort of children with CH was stunted: 33.3% (n=22) were moderately stunted (HAZ < –2SD) and 19.7% (n=13) severely stunted (HAZ < –3SD) (p < 0.001). Similarly, 36.4% (n=24) were underweight, including 21.2% (n=14) with moderate (WAZ < –2SD) and 15.2% (n=10) with severe underweight (WAZ < –3SD). Among 35 stunted children, 32 (91.4%) had extrapolated adult heights below their mid-parental height (MPH) target. BMI was lower in children with CH than controls, although the difference did not reach statistical significance (**Table 2**). Head circumference Z-scores also reflected growth impairment. In boys, mean values improved from –1.31 at age 1 to –0.68 at age 5; in girls, from –0.85 to –0.09 (**Table 1**). Eight children (12.1%) had small heads (HC-for-age < –2SD), and six (9.1%) had microcephaly (HC-for-age < –3SD), indicating significant deviations in head growth (p < 0.001).

**Table 1:** Comparison of anthropometric Z-scores among children with congenital hypothyroidism and healthy controls, stratified by age and sex (1–5 years).

Children with CH																					
	HAZ			WAZ			BMI for age Z score			Head Circumference for age Z score			MUAC for age Z score			Triceps SFT for age Z score			Sub-scapular SFT for age Z score		
	M	F	GD	M	F	GD	M	F	GD	M	F	GD	M	F	GD	M	F	GD	M	F	GD
Age	Mean ± SD (CI)	Mean ± SD	p-value	Mean ± SD (CI)	Mean ± SD (CI)	p-Value	Mean ± SD (CI)	Mean ± SD (CI)	p-Value	Mean ± SD (CI)	Mean ± SD (CI)	p-Value	Mean ± SD (CI)	Mean ± SD (CI)	p-Value	Mean ± SD	Mean ± SD (CI)	p-Value	Mean ± SD (CI)	Mean ± SD (CI)	p-Value
1	-2.77 ± 1.36 (-4.19, -1.34)	-2.94 ± 1.11 (-4.11, -1.77)	0.63	-1.90 ± 1.11 (-3.06, -0.74)	-2.22 ± 1.27 (-3.56, -0.89)	0.748	-0.32 ± 1.41 (-1.80, 1.15)	-0.60 ± 1.01 (-1.66, 0.47)	0.63	-1.31 ± 0.52 (-1.85, -0.77)	-0.85 ± 1.36 (-2.28, 0.58)	0.521	-0.55 ± 1.47 (-2.10, 1.00)	-1.02 ± 1.32 (-2.41, 0.36)	0.63	0.52 ± 1.65 (-1.21, +2.26)	-0.80 ± 1.40 (-2.27, 0.67)	0.078	0.33 ± 1.87 (-1.64, +2.29)	-1.09 ± 1.29 (-2.44, 0.27)	0.465
2	-1.64 ± 0.99 (-2.31, -0.98)	-1.80 ± 0.80 (-3.79, 0.18)	0.755	-0.39 ± 1.25 (-1.23, +0.45)	-0.96 ± 0.26 (-1.61, -0.31)	0.392	0.89 ± 1.32 (0.01, 1.78)	0.20 ± 0.55 (-1.16, 1.56)	0.312	-0.86 ± 1.73 (-2.02, 0.30)	0.59 ± 0.44 (-0.50, 1.67)	0.052	-0.35 ± 1.00 (-1.03, 0.32)	-0.80 ± 0.31 (-1.57, -0.03)	0.139	1.35 ± 1.04 (0.65, 2.04)	1.11 ± 0.87 (-1.05, 3.28)	0.697	1.09 ± 1.16 (0.31, 1.87)	0.38 ± 0.47 (-0.79, 1.56)	0.011*
3	-2.59 ± 2.45 (-4.47, -0.71)	-2.16 ± 0.47 (-2.91, -1.40)	1	-2.21 ± 2.48 (-4.12, -0.30)	-1.53 ± 0.51 (-2.34, -0.71)	0.758	-0.91 ± 1.51 (-2.07, 0.25)	-0.17 ± 0.62 (-1.15, 0.81)	0.44	-2.00 ± 1.97 (-3.51, -0.49)	-1.51 ± 1.62 (-4.09, 1.07)	0.758	-1.79 ± 1.50 (-2.94, -0.64)	-1.01 ± 0.94 (-2.51, 0.49)	0.44	0.69 ± 1.18 (-0.22, 1.60)	0.32 ± 0.90 (-1.11, 1.76)	0.537	-0.26 ± 1.41 (-1.34, 0.83)	0.49 ± 0.28 (0.04, 0.94)	1
4	-2.56 ± 1.71 (-3.79, -1.34)	-2.18 ± 2.47 (-5.25, 0.88)	0.624	-2.28 ± 1.85 (-3.60, -0.95)	-1.77 ± 1.41 (-3.52, -0.01)	0.462	-0.90 ± 1.55 (-2.01, 0.21)	-0.39 ± 0.75 (-1.32, 0.53)	0.54	-1.0.2 ± 1.51 (-2.10, 0.06)	-0.64 ± 0.59 (-1.37, 0.10)	0.806	-1.57 ± 1.09 (-2.36,.0.79)	-1.46 ± 0.41 (-1.97, -0.95)	0.624	0.22 ± 1.13 (-0.59, 1.03)	-0.21 ± 0.78 (-1.18, 0.75)	0.462	0.66 ± 0.73 (0.13, 1.18)	-0.62 ± 1.13 (-2.02, 0.77)	0.178
5	-1.91 ± 1.65 (-3.44, -0.38)	-0.96 ± 0.68 (-1.81, – 0.11)	0.57	-1.27 ± 1.01 (-2.21, -0.34)	-0.89 ± 0.78 (-1.87, 0.08)	0.465	0.02 ± 1.05 (-0.95, 0.99)	-0.47 ± 0.53 (-1.12, 0.18)	0.57	-0.68 ± 0.80 (-1.41, 0.06)	-0.09 ± 1.32 (-1.72, 1.55)	0.088	-0.54 ± 1.03 (-1.49, 0.41)	-0.59 ± 0.68 (-1.44, 0.25)	0.465	0.68 ± 1.42 (-0.64, 1.99)	0.59 ± 0.86 (-0.48, 1.65)	0.685	1.09 ± 1.74 (-0.52, 2.70)	0.86 ± 1.12 (-0.53, 2.25)	0.245

Healthy Controls																					
	HAZ			WAZ			BMI for age Z score			Head Circumference for age Z score			MUAC for age Z score			Triceps SFT for age Z score			Sub-scapular SFT for age Z score		
	M	F	GD	M	F	GD	M	F	GD	M	F	GD	M	F	GD	M	F	GD	M	F	GD
Age	Mean ± SD (CI)	Mean ± SD (CI)	p-Value	Mean ± SD (CI)	Mean ± SD (CI)	p-Value	Mean ± SD (CI)	Mean ± SD (CI)	p-Value	Mean ± SD (CI)	Mean ± SD (CI)	p-Value	Mean ± SD (CI)	Mean ± SD (CI)	p-Value	Mean ± SD (CI)	Mean ± SD (CI)	p-Value	Mean ± SD (CI)	Mean ± SD (CI)	p-Value
1	-0.76 ± 0.99 (-1.7,0.15)	-1.01 ± 0.86 (-2.07,0.05)	0.57	-0.53 ± 1.03 (-1.48,0.41)	-0.81 ± 0.54 (-1.47,-0.14)	0.935	-0.12 ± 1.14 (-1.17,0.93)	-0.30 ± 0.78 (-1.27,0.66)	0.808	-0.58 ± 1.00 (-1.50,0.34)	-0.52 ± 1.35 (-2.19,1.15)	0.935	-0.22 ± 0.85 (-1.0,0.56)	-0.56 ± 0.95 (-1.73,0.61)	0.57	1.03 ± 1.07 (0.4,2.02)	0.99 ± 0.62 (0.22,1.76)	0.685	1.15 ± 0.70 (0.51,1.79)	0.91 ± 0.53 (0.25,1.56)	0.465
2	-0.41 ± 0.66 (-1.23,0.41)	-0.47 ± 0.88 (-1.39,0.56)	0.855	0.68 ± 1.24 (-0.85,2.21)	-0.66 ± 0.88 (-1.58,0.25)	0.1	1.27 ± 1.55 (-0.65,3.12)	-0.59 ± 0.91 (-1.54,0.36)	0.1	-0.84 ± 0.95 (-2.01,0.34)	-0.79 ± 0.66 (-1.47,-0.09)	0.584	0.64 ± 1.29 (-0.96,2.24)	-0.30 ± 0.42 (-0.60,2.27)	0.1	1.33 ± 0.64 (1.01,2.58)	1.43 ± 0.54 (0.86,2.00)	0.465	1.31 ± 0.78 (0.29,2.23)	0.72 ± 0.53 (-0.46,0.64)	0.011*
3	-0.65 ± 1.06 (-1.44,0.32)	-0.55 ± 0.47 (-1.71,0.52)	0.15	0.04 ± 1.07 (-0.86,0.93)	-0.24 ± 0.35 (-1.87,0.30)	0.794	0.56 ± 0.91 (-0.19,1.32)	-0.20 ± 0.95 (-0.78,0.25)	0.296	-0.76 ± 0.62 (-1.28, -0.24)	-0.61 ± 0.12 (-1.72,0.49)	0.794	0.12 ± 1.26 (-0.88,1.22)	-0.11 ± 1.64 (-0.40,0.35)	0.433	1.03 ± 0.92 (0.79,2.34)	0.85 ± 1.65 (0.67,2.89)	0.433	1.28 ± 0.52 (0.84,1.71)	1.11 ± 0.85 (0.29,2.27)	1
4	-0.76 ± 0.76 (-1.16, -0.35)	-0.20 ± 0.84 (-0.90,0.49)	0.086	-0.76 ± 0.65 (-1.10, -0.41)	-0.68 ± 0.81 (-1.35,0.01)	0.927	-0.34 ± 0.53 (-0.62, -0.05)	-0.86 ± 0.91 (-1.61, -0.10)	0.198	-1.05 ± 0.46 (-1.29, -0.80)	-0.88 ± 0.51 (-1.31, -0.45)	0.391	-0.72 ± 0.66 (-1.07, -0.36)	-0.88 ± 0.69 (-1.45, -0.30)	0.582	0.80 ± 0.87 (0.33,1.26)	-0.07 ± 0.86 (-0.79,0.64)	0.037*	0.60 ± 0.87 (0.15,1.04)	0.21 ± 0.81 (-0.46,0.89)	0.178
5	-0.30 ± 1.00 (-0.96, -0.40)	-0.43 ± 0.58 (-1.14,0.29)	0.699	-0.34 ± 0.03 (-0.59, -0.08)	-0.38 ± 1.03 (-1.66,0.91)	0.699	-0.30 ± 0.97 (-0.69,1.10)	-0.21 ± 1.09 (-1.55,1.14)	0.699	-0.17 ± 0.54 (-1.40, -0.30)	-0.01 ± 0.87 (-1.08, -1.07)	0.699	-0.33 ± 0.17 (-1.85,1.19)	-0.95 ± 0.50 (-1.56, -0.33)	0.121	-0.76 ± 0.06 (-1.32, -0.80)	0.33 ± 0.61 (-0.43,1.08)	0.053	-0.01 ± 0.28 (-0.20,1.23)	0.51 ± 0.58 (-0.21,1.12)	0.245

CI, Confidence Interval; GD, Gender Difference; M, Male; F, Female, *p ≤ 0.05.

**Table 2:** Intergroup comparison of anthropometric Z-scores between children with congenital hypothyroidism (CH) and healthy controls, stratified by age (1–5 years) and sex.

Male							
Age	HAZ	WAZ	BMI for age Z score	HC for age Z score	MUAC for Age Z score	Triceps SFT for age Z score	Sub Scapular SFT for age Z score
1	0.003**	0.086	0.775	0.116	0.475	0.317	0.253
2	0.02*	0.157	0.396	0.533	0.079	0.336	0.692
3	0.248	0.054	0.027*	0.29	0.034*	0.178	0.009**
4	0.001**	0.004**	0.246	0.206	0.031*	0.206	0.712
5	0.242	0.04*	0.77	0.558	0.558	0.143	0.558

Female							
Age	HAZ	WAZ	BMI for age Z score	HC for age Z score	MUAC for Age Z score	Triceps SFT for age Z score	Sub Scapular SFT for age Z score
1	0.017*	0.044*	1	0.714	0.583	0.067	0.006**
2	0.071	1	0.197	0.039*	0.071	0.439	0.439
3	0.064	0.064	0.643	0.355	0.643	0.643	0.165
4	0.079	0.143	0.306	0.242	0.079	0.558	0.188
5	0.347	0.917	0.917	0.917	0.251	0.602	0.347

*p ≤ 0.05, **p ≤ 0.01.

Growth deficits extended to MUAC, skinfold thickness (SFT), upper segment, and arm span, all of which were lower in children with CH than controls. Based on WHO 2006 MUAC-for-age standards, 10 children (15.2%) had moderate acute malnutrition (< –2SD), and 3 (4.5%) had severe acute malnutrition (< –3SD) (p<0.001). Triceps and subscapular SFTs were consistently lower in children with CH, with significantly reduced values noted at age 3 (p= 0.012), suggesting persistent deficits in subcutaneous fat deposition (**Tables 1**, **2**).

### Proportionality parameters

To assess proportionality of stature in children with CH, anthropometric measures included the difference between height and arm span, and the Upper Segment to Lower Segment (US: LS) ratio. Assessment of height–arm span difference revealed a significantly higher prevalence of disproportionate stature in children with CH compared to controls. Disproportion-defined as a difference greater than ±3.5 cm—was observed in 56.1% of children with CH, whereas all controls demonstrated normal body proportions (p < 0.0001). The US: LS ratio was also elevated in children with CH, particularly at younger ages, reflecting infantile body proportions. At 1 year of age, mean US: LS ratios in CH boys and girls were 1.83 and 1.70, respectively, compared to 1.49 in age-matched controls. By 5 years, these values declined to 1.29 in boys and 1.19 in girls, approaching normative values of 1.16 and 1.17. Overall, 44 children with CH (66.7%) exhibited infantile proportions based on US: LS, while all controls showed age-appropriate ratios (p < 0.0001). Disproportionate growth was significantly more common among children with CH with greater stunting severity. Based on height–arm span difference, 68.2% of moderately stunted and 100% of severely stunted children had disproportionate short stature. Similarly, infantile proportions based on US: LS ratio were seen in 90.9% of moderately and 76.9% of severely stunted children with CH (**Table 3**). These findings demonstrate a strong and statistically significant association between increasing severity of linear growth failure (lower HAZ) and altered body proportions, as assessed by both height–arm span difference (p < 0.0001) and US: LS ratio (p = 0.002), while all controls exhibited normal body proportions.

**Table 3:** Body proportions of children with congenital hypothyroidism as estimated by difference of height (cm) and arm span (cm); and US: LS ratio.

	Difference of Height-Arm span		US: LS Ratio	
HAZ Category	Proportionate	Disproportionate	Proportionate	Disproportionate
Normal	22 (71.0%)	9 (29.0%)	17 (54.8%)	14 (45.2%)
Moderate Stunting	7 (31.8%)	15 (68.2%)	2 (9.1%)	20 (90.9%)
Severe Stunting	0	13 (100%)	3 (23.1%)	10 (76.9%)
p-value	<0.0001***		0.002**	

**p ≤ 0.01, ***p ≤ 0.0001.

The Manouvrier’s Indice Skélique (MIS), used to assess body proportionality, was consistently lower in children with CH compared to healthy controls. The difference was statistically significant among boys at all ages (p < 0.05) and among girls at 1, 2, and 4 years of age (p < 0.05). Based on MIS classification, none of the children with CH fell into the macroskelia or mesatyskelia categories. Instead, all 66 children with CH were classified as having brachyskelia, reflecting disproportionately short legs relative to trunk length.

### Impact of treatment timing

The timing of initiation of LT4 therapy in CH plays a critical role in determining long-term growth and body proportionality. To assess this, children with CH were categorized into two groups based on age at LT4 initiation: early treatment group (<3 months, n=35) and late treatment group (>3 months, n=31). Although mean height-for-age (HAZ), weight-for-age (WAZ), and head circumference-for-age (HCZ) were higher in the early treatment group, these differences were not statistically significant. Severe stunting (HAZ < –3 SD) was significantly more prevalent in the late treatment group (67.7% vs. 11.4%; p < 0.05), with all severely stunted participants having height below their genetic potential. Likewise, severe underweight (WAZ < –3 SD) was significantly more common in the late group (29% vs. 2.9%; p < 0.05), as was microcephaly or small head circumference (HCZ < –2 SD) (35.5% vs. 8.6%; p < 0.05). These results underscore the compounded impact of delayed therapy on both linear and cerebral growth. Indicators of nutritional status, MUAC, SFTs, and BMI, were consistently higher in the early treatment group.

Assessment of body proportionality using the height-to-arm span ratio revealed that disproportionate stature was significantly more prevalent in the late treatment group (71.0%, *n* = 22) compared to the early treatment group (42.9%, *n* = 15; *p* < 0.05). Complementing this, evaluation based on the upper segment-to-lower segment (US: LS) ratio demonstrated a predominance of infantile body proportions in both groups, with a higher frequency observed in the late-treated group (74.2%) compared to the early-treated group (60%), although this difference was not statistically significant (p = 0.222).

When stratified by the degree of stunting, disproportionate stature-as assessed by the difference between height and arm span-was observed in 80% of moderately stunted children in the early treatment group and 58.3% in the late group. All severely stunted children (100%) in both early and late treatment groups exhibited disproportionate short stature. This association was statistically significant in the early treatment group (*p* < 0.0001). A similar trend was observed with the upper segment-to-lower segment (US: LS) ratio, where infantile proportions were present in 90% of moderately and 75% of severely stunted children in the early treatment group, compared to 91.7% and 77.8%, respectively, in the late treatment group. These differences were statistically significant within the early treatment group (*p* = 0.035), indicating a strong association between the severity of stunting and the presence of body disproportionality.

Collectively, these findings demonstrate that delayed initiation of LT4 therapy beyond 3 months is associated with significantly higher rates of stunting, underweight, microcephaly, and disproportionate body proportions. Despite some gains in growth parameters, the adverse effects of late therapy persist and appear to be partially irreversible.

## Discussion

Congenital hypothyroidism (CH) is one of the most prevalent endocrine disorders in pediatric populations, profoundly influencing growth and body proportionality. The absence of a universal NBS program for CH in India, combined with its subtle clinical presentation, often leads to delays in diagnosis and treatment initiation. These delays are well-documented to result in short stature, however the degree and proportionality of growth deficits remain contentious. This study evaluates the impact of CH and delayed LT4 therapy initiation on various anthropometric parameters. The mean age at diagnosis for children with CH in this study was 3.42 ± 3.44 months, with a median age of 2 months. This delay is substantial compared to countries with established NBS programs, where diagnosis occurs within 10–14 days, as recommended in various guidelines (28, 29, 34, 35). The study population showed a male predominance among children with CH, contrasting with the global trend of slight female predominance. Cultural and socio-economic factors in resource-limited settings like ours, may prioritize healthcare access for male children, potentially explaining this gender disparity. The control group also showed a male predominance, though less pronounced than in the CH group (36).

**Figure 1:**
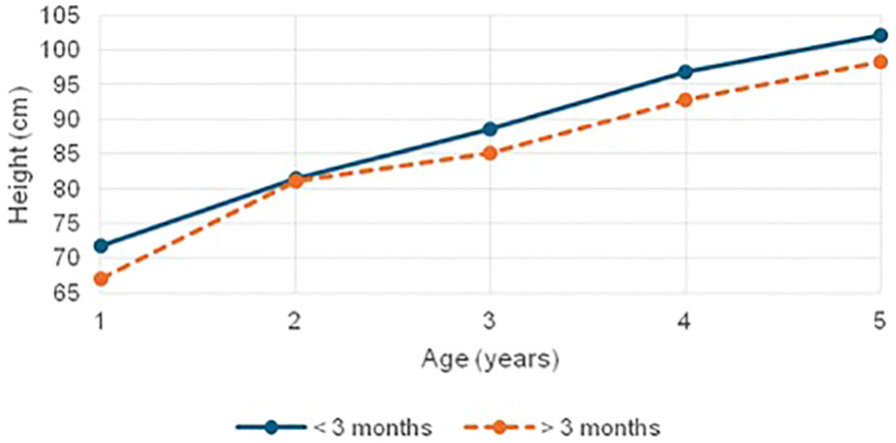
Mean height comparison in children with CH based on age at treatment initiation (< 3 Months vs > 3 Months).

**Figure 2:**
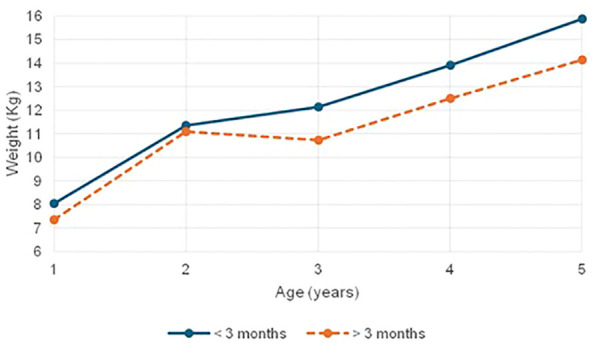
Mean weight comparison in children with CH based on age at treatment initiation (< 3 Months vs > 3 Months).

Children with CH exhibited consistently lower anthropometric measures: height, weight, and head circumference as compared to controls. Those initiated on treatment early showed relatively preserved height, while delayed treatment was associated with significantly greater and more severe growth faltering (**Figure 1**). Importantly, all severely stunted children in the late-treated group had heights below their genetic potential, underscoring the compounded effect of delayed treatment on linear growth and emphasizing the importance of timely intervention. Consistent with these findings, retrospective cohort data indicate that even with timely diagnosis and appropriate LT4 therapy, a subset of children with CH may exhibit impaired linear growth; additionally, evidence from population- and target height–referenced analyses suggests incomplete catch-up growth relative to genetic height potential (37, 38). Weight parameters were significantly lower in children with CH compared to controls, with girls showing more pronounced deficits. In our cohort, early-treated children had relatively preserved weight, whereas those treated later showed significant weight faltering, a trend that was statistically significant (p = 0.003) (**Figure 2**). The findings echo those of Feizi et al. (19), who reported that untreated hypothyroidism impairs not only height but also weight gain due to under treatment, which was certainly not the case in our cohort. We attribute the growth delay to the delayed diagnosis in our children. Heidari et al. (21) also reported that delayed initiation of treatment results in significant stunting and impaired height and weight outcomes as thyroid hormones play a vital role in growth plate activity during the period of rapid growth in early infancy. Nutritional indicators such as MUAC Z-scores and skinfold thickness were also significantly lower in children with CH, reflecting compromised fat stores and nutritional status. Interestingly, BMI Z-scores in children with CH showed limited variability and remained largely within normal limits, with only a small proportion (10.6%) falling below –2 SD. These findings are in line with studies by Kyo Ha (39) and Nazari (40), who suggested that while linear growth may be impaired, BMI often remains within normal limits. Kyo Ha et al. attributed this to normal weight gain compensating for poor height gain, a pattern not observed in our study population (39). Instead, the normal BMI in our children with CH was due to concurrent faltering in both height and weight, effectively masking underlying growth deficits. Similarly, Nazari et al. (40) linked normal BMI to early diagnosis and treatment through Iran’s NBS program, along with effective LT4 therapy ensuring normal metabolism and regular follow-ups. This explanation does not align with our findings either. These findings underscore that BMI may not reliably reflect nutritional status or growth adequacy in CH, as it can obscure the dual impact of thyroid hormone deficiency on both stature and body mass. This is further supported by Feizi et al. (19), who described metabolic slowing in untreated CH leading to reduced lean mass and energy reserves.

**Figure 3:**
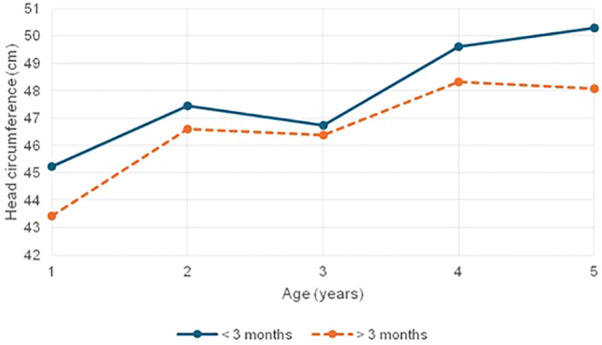
Mean head circumference comparison in children with CH based on age at treatment initiation (< 3 Months vs > 3 Months).

Neurocranial development, as reflected by head circumference, was significantly compromised in children with CH with delayed treatment initiation (**Figure 3**), underscoring the critical role of thyroid hormones in early brain and skull growth. The higher prevalence of microcephaly and small head size in the late-treated group reinforces this association. Contrary to Van Vliet et al.’s observation of transient macrocrania due to delayed skeletal maturation, our findings did not reveal such a trend; instead, early therapy appeared to support more preserved head growth, aligning with previous observations by Alinia et al. and Nazari et al. linking reduced HC to delayed treatment and more severe hypothyroidism (40–42).

**Figure 4:**
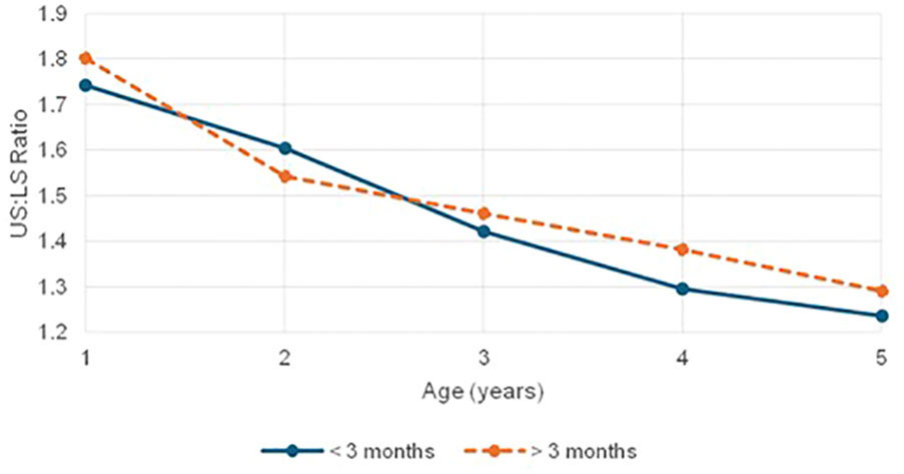
Mean US . LS ratio comparison in children with CH based on age at treatment initiation (< 3 Months vs > 3 Months).

**Figure 5:**
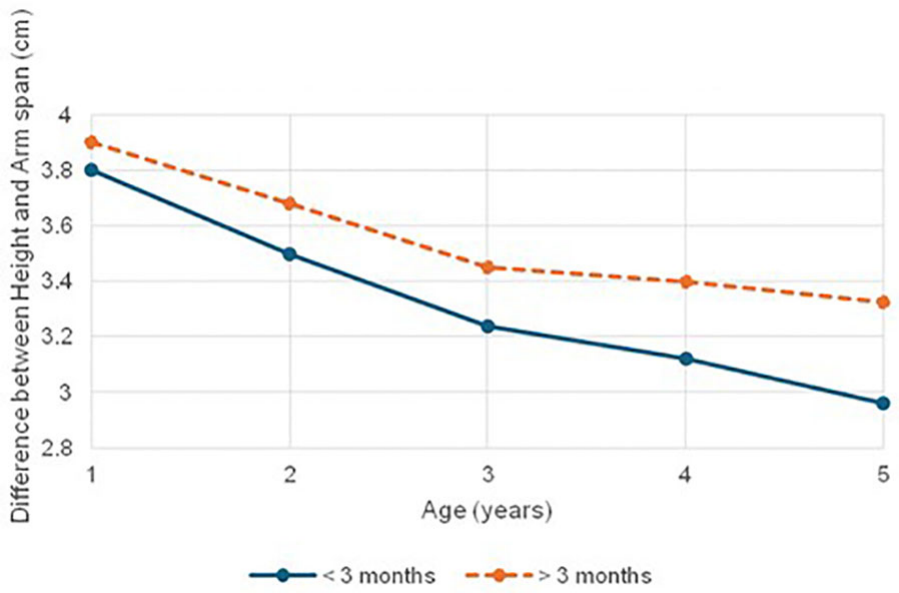
Mean height-arm span comparison in children with CH based on age at treatment initiation (< 3 Months vs > 3 Months).

In addition to cranial growth, a distinct pattern of disproportionate somatic growth was noted, with nearly two-thirds (67%) of children with CH displaying infantile body proportions, particularly among younger and severely stunted participants. Elevated US: LS ratios across age groups suggest relatively preserved trunk growth relative to limbs, indicating that thyroid hormone deficiency disrupts harmonious development of body segments. This disproportionality was more frequent and pronounced in the late-treated group, underscoring the cumulative impact of delayed therapy (**Figures 4**, **5**). Supporting this, height–arm span discrepancies were observed in 56.1% of the cohort, especially among the severely stunted, where altered body proportions were nearly universal. These findings are consistent with earlier studies by Singh et al., Gutch et al., and Ramgopal et al., which attributed such patterns to delayed skeletal maturation (43–45). By linking disproportionality to treatment timing and stunting severity, our study reinforces that thyroid hormone deficiency impairs not only overall growth but also skeletal proportion. These disruptions likely reflect underlying abnormalities in growth plate dynamics and ossification. The results emphasize the need for early diagnosis and treatment, along with routine anthropometric assessments including proportionality indices to detect subtle yet clinically significant skeletal impairments.

Our study demonstrates that initiation of LT4 therapy within the first three months of life is critical for preserving linear growth, cranial development, and skeletal proportionality in children with CH. Delayed treatment was associated with adverse effects on limb growth, resulting in persistent alterations in body proportions, including increased US: LS ratios and truncal predominance favoring infantile proportions. These abnormalities, once established during early infancy, may not fully normalize despite later initiation of therapy, highlighting the presence of sensitive developmental windows for skeletal growth. Importantly, growth impairment in CH extends beyond deficits in height alone, underscoring the limitations of relying solely on linear growth indices during follow-up. Incorporation of body proportionality and segmental growth assessments into routine clinical monitoring may facilitate earlier identification of persistent growth disturbances. These findings reinforce the essential role of universal NBS and timely LT4 therapy in promoting harmonious skeletal development and optimizing long-term growth outcomes.

### Limitations

Several considerations should be acknowledged when interpreting these findings. The cross-sectional study design precludes evaluation of longitudinal growth trajectories. As this was a single-center study, the results may be influenced by local referral patterns and may limit generalizability. In addition, the present analysis was confined to growth and body proportionality outcomes; neurodevelopmental, psychosocial, and quality-of-life measures were beyond the scope of this investigation.

### Future directions

Prospective, longitudinal studies with larger, multicentric cohorts are needed to better characterize growth trajectories and body proportionality in children with congenital hypothyroidism. Inclusion of detailed early-life anthropometric data and extended follow-up would help clarify the reversibility of growth and proportionality deficits associated with delayed treatment.

## Conclusion

Children with CH exhibited significant impairments in linear growth, nutritional status, neurocranial development, and body proportionality compared with healthy peers. Initiation of LT4 therapy beyond three months of age was associated with markedly higher prevalence of severe stunting, underweight, microcephaly, and disproportionate short stature, accompanied by persistent infantile body proportions. These findings highlight the critical importance of early diagnosis and timely initiation of treatment for CH and provide strong support for universal NBS programs in India to optimize growth outcomes and preserve normal body proportionality.

## Data Availability

The original contributions presented in the study are included in the article/supplementary material. Further inquiries can be directed to the corresponding author.
